# Pro-neural transcription factors as cancer markers

**DOI:** 10.1186/1755-8794-1-17

**Published:** 2008-05-19

**Authors:** Maria Vias, Charlie E Massie, Philip East, Helen Scott, Anne Warren, Zongxiang Zhou, Alexander Yu Nikitin, David E Neal, Ian G Mills

**Affiliations:** 1 Uro-Oncology Research Group, Cancer Research UK Cambridge Research Institute, Li Ka Shing Centre, Robinson Way, Cambridge, CB2 0RE, UK; 2 Bioinformatics & Biostatistics, Cancer Research UK London Research Institute, 44 Lincoln's Inn Fields, London, WC2A 3PX, UK; 3 Department of Pathology, Addenbrookes Hospital, Cambridge, CB2 2QQ, UK; 4 Department of Biomedical Sciences, Cornell University, Ithaca, New York, 14853, USA

## Abstract

**Background:**

The aberrant transcription in cancer of genes normally associated with embryonic tissue differentiation at various organ sites may be a hallmark of tumour progression. For example, neuroendocrine differentiation is found more commonly in cancers destined to progress, including prostate and lung. We sought to identify proteins which are involved in neuroendocrine differentiation and differentially expressed in aggressive/metastatic tumours.

**Results:**

Expression arrays were used to identify up-regulated transcripts in a neuroendocrine (NE) transgenic mouse model of prostate cancer. Amongst these were several genes normally expressed in neural tissues, including the pro-neural transcription factors Ascl1 and Hes6. Using quantitative RT-PCR and immuno-histochemistry we showed that these same genes were highly expressed in castrate resistant, metastatic LNCaP cell-lines. Finally we performed a meta-analysis on expression array datasets from human clinical material. The expression of these pro-neural transcripts effectively segregates metastatic from localised prostate cancer and benign tissue as well as sub-clustering a variety of other human cancers.

**Conclusion:**

By focussing on transcription factors known to drive normal tissue development and comparing expression signatures for normal and malignant mouse tissues we have identified two transcription factors, Ascl1 and Hes6, which appear effective markers for an aggressive phenotype in all prostate models and tissues examined. We suggest that the aberrant initiation of differentiation programs may confer a selective advantage on cells in all contexts and this approach to identify biomarkers therefore has the potential to uncover proteins equally applicable to pre-clinical and clinical cancer biology.

## Background

In recent years there has been much effort to identify new prostate cancer biomarkers. Malignant prostatic tumours commonly contain scattered or focal neuroendocrine type cells, but only a small minority or prostate cancers contain an homogenous population of such cells, when they are classified as small cell prostatic carcinoma. However, other regular prostate carcinomas which have an increased NE phenotype are at increased risk of tumour progression and castration resistance [1-3]. We recently reported that long-term anti-androgen treatment induces NE differentiation in a cell line model, giving rise to a more invasive phenotype [4].

Some previous studies have failed to find convincing correlations between focal NE differentiation and prostate cancer progression [5-7] Variations in expression and detection of neuron-specific enolase, chromogranin A and synaptophysin may be partly responsible for this controversy. Therefore better markers for a neural or neuroendocrine phenotype would benefit the field.

Multiple basic helix-loop-helix (bHLH) proteins play a critical role in the regulation of neural stem cell differentiation [8]. The bHLH family of transcription factors includes activators and repressors of transcription. The activator-type bHLH transcription factors include 'achaete-scute complex' homologue 1 (Ascl1) which is expressed in differentiating neurons and belongs to the Neurogenin Family. This activating bHLH transcription factor is believed to drive the expression of a 'hairy and enhancer of split' factor, Hes6. Hes6 in turn can support Ascl1 activity and neuronal differentiation in part by antagonising Hes1 activity through heterodimer formation [9]. Hes1 is a repressor-type bHLH transcription factor which maintains neural stem cells by repressing activator bHLH expression [10]. In the case of Hes1 this occurs at two levels: firstly through direct binding to the Ascl1 promoter, and secondly by forming a non-functional heterodimer with another activator-type bHLH transcription factor, E47 [9,11]. Overall, Hes proteins are involved in the maintenance of neural stem cells and gliogenesis, whilst Ascl1 is implicated in neurogenesis [12-14].

## Methods

### In silico approaches

Expression array data from *p53*^*PE*-/-^; *Rb*^*PE*-/- ^cancerous (n = 5) and normal (n = 3) samples were retrieved from a previously published data set [15]. Gene expression data from the *p53*^*PE*-/-^; *Rb*^*PE*-/- ^mouse model expression array data were analysed in the R statistical software using the limma and affy packages [16,17]. Briefly, data were pre-processed using the RMA (Robust Multichip Average) method, before fitting a linear model and applying Bayesian smoothing to identify differentially expressed genes between the normal and cancer samples. M-values (log2 expression ratios) were calculated for all probes and for each sample and then complete hierarchical clustering was performed using the Eisen Cluster program [18]. Heatmaps were generated using the Eisen TreeView program.

Median centred log2 ratios of normal adult tissue transcript levels were retrieved from the Oncogenomics Normal Tissue Database [19] for genes which were found to be differentially regulated in the *p53*^*PE*-/-^; *Rb*^*PE*-/- ^mouse model of prostate cancer using IMAGE clone identifiers retrieved from the Clone/Gene ID converter [20,21].

Clinical prostate cancer expression array data were retrieved from the NCBI Gene Expression Omnibus (accession numbers GSE3325 and GSE6099) from a previously published Affymetrix expression array data set. To generate dot plots, data were pre-processed using the RMA (Robust Multichip Average) method, quantile normalised and intensity estimate values were averaged for all probes for a given gene.

Clinical cancer expression array data sets (ExpO) covering 1786 multi-tissue tumour specimens were retrieved from the NCBI Gene Expression Omnibus (accession number GSE2109). Preprocessed MAS5 intensity estimates were median-centred for each chip and each gene scaled to its median intensity value across all samples. Log2 neuroendocrine biomarker expression vectors across all 1786 samples were extracted (*Hes6, Ascl1, Chga, Ddc, Nts *and *Pou5f1*). These are displayed as a heatmap. Genes were functionally grouped (*Hes6, Hes6-Chga, Hes6-Ddc, Ascl1, Ascl1-Hes6-Nts-Ddc*) and a median expression intensity calculated per group for each sample. Samples showing a greater than 3 fold increase in expression per group were selected. Selected samples are highlighted by black bars above the heatmap. Samples assigned to each gene group were tested for enrichment of malignant tissue type when compared to the complete dataset using a hyper-geometric distribution. Enriched tumour types (p-value < 0.05) are shown in Table 1 and are highlighted as coloured bars on the heatmap. The analysis was carried out within R using Bioconductor [16].

**Table 1:** Statistical significance (p values) for a panel of neuroendocrine markers in human tumours.

**Gene**	**BN vs PCa**	**BN vs MET**	**PCa vs MET**
**Ascl1**	0.296481304	0.003042	0.00154356
**Hes6**	0.024247479	0.01678206	0.01129449
**Nts**	0.472811274	0.05652598	0.03387797
**Nse**	0.001424731	0.03568144	0.01538955
**Chga**	0.102607964	0.0783078	0.05591173
**AR**	0.209422779	0.05160438	0.04404816
**Hes1**	0.041737561	0.16434413	0.40890498
**NeuroD1**	0.210143163	0.10369964	0.06466747
**Syt4**	0.00753976	0.04080721	0.00655119
**Asph**	0.241306154	0.00261299	0.00148242
**Atp11a**	0.059113322	0.0058551	0.27651832
**Ddc**	0.307964378	0.01022954	0.00378398
**Ngn1**	0.266250502	0.28859185	0.4960235
**Ngn2**	0.069344987	0.06267247	0.03905491
**Ngn3**	0.270696485	0.03998213	0.08993646

Significance values were generated by comparing the fold-change in gene expression between expression levels in the dataset of Varambally et al., (2005) and examining benign (BN), localised prostate cancer (PCa) and metastatic (MET) samples. A two sample Student's t-Test with a one-tailed distribution was used to obtain the p-values.

### Statistical Analysis of the ExpO Dataset

Each chips MAS5 generated signal intensity estimates were scaled to the chip median. We acknowledge a quantification algorithm such as RMA would be a preferable step here but due to the large size of the data set and available computational resources this proved problematic. The normalised expression vectors and sample annotation were databased using MySQL to allow efficient access to the expression profiles [22]. The DBI package within R was used to extract neuroendocrine gene profiles (205311_at:DDC, 206291_at:NTS, 206940_s_at:POU4F1, 209985_s_at:ASCL1, 209987_s_at:ASCL1, 209988_s_at:ASCL1, 211341_at:POU4F1, 213768_s_at:ASCL1, 214347_s_at:DDC, 226446_at:HES6, 228169_s_at:HES6, 204697_s_at:CHGA). Each gene profile was scaled to its median value across all samples and a Bioconductor Expression Set object created. The profiles were grouped into functional gene sets and a median signal intensity calculated per sample (*Hes6*, *Hes6-Chga*, *Hes6-Ddc*, *Ascl1*, *Ascl1-Hes6-Nts-Ddc*). Samples displaying a greater than 3 fold induction were selected. Identified samples were then tested for enrichment of tumour type using a hyper-geometric distribution. (R code provided below). The function phyper.expo(decide, pdat, th = 2) is called with a vector (decide) indicating up regulated, down regulated and unselected genes (1, -1, and 0 respectively), a dataframe (pdat) containing tumour type labels etc. as columns and a threshold (tr) setting the minimum number of tumour type hits within the selected samples to report. A list is returned containing tumour label, the number of tumour type samples within the sample set (hits), the number of tumour type samples within the ExpO dataset,(foreground), the number of non tumour type samples within the ExpO dataset (background), the number of samples selected on expression (sample.size) and the p-value (p. value) associated with enrichment.

### Quantitative real time polymerase chain reaction (qRT-PCR)

RNA extraction, cDNA synthesis and qRTPCR were performed as previously described [4]. Primers for quantitative real time polymerase chain reaction using Sybrgreen chemistry were designed using Primer Express software (Applied Biosystems) (Table 2). Another set of primer sequences were used as previously reported like dopa decarboxylase [23], NeuroD1 and Neurogenin 2 [24], Hes1 [25], Hes6 [26] and Ascl1 [27].

**Table 2:** Primer sequences for quantitative real-time PCR validation.

**Gene**	**Primer sense**	**Primer antisense**
SDH	TGGGAACAAGAGGGCATCTG	CCACCACTGCATCAAATTCATG
GAPDH	GCAAATTCCATGGCACCGT	TCGCCCCACTTGATTTTGG
UBC	ATTTGGGTCGCGGTTCTTG	TGCCTTGACATTCTCGATGGT
TBP	GAATATAATCCCAAGCGGTTTG	ACTTCACATCACAGCTCCCC
Neuromedin/NT	GAGGAGCTTGTTGCAAGAAGGA	GCCCTGCTGTGACAGATTTTGT
Hes6	AGCTCCTGAACCATCTGCTC	GACTCAGTTCAGCCTCAGGG
Ascl1	GAGCAGGAGCTTCTCGACTTC	GATGCAGGTTGTGCGATCAC
Hes1	TGGAAATGACAGTGAAGCACCT	GTTCATGCACTCGCTGAAGC
NeuroD1	CGCTGGAGCCCTTCTTTG	GCGGACGGTTCGTGTTTG
DDC	AAGCACAGCCATCAGGATTCA	TGGACATGCTTGCGGATATAAG
Neurogenin 2	CGCATCAAGAAGACCCGTAG	GTGAGTGCCCAGATGTAGTTGTG

Primers for quantitative real time polymerase chain reaction using Sybrgreen chemistry were designed using Primer Express software (Applied Biosystems)

### Small interference RNA silencing

Cells were washed with PBS, trypsinized and centrifuged at 1300 rpm for 3 minutes. The cell pellet was resuspended and cells were counted using the haemocytometer. 2 × 10^6 ^cells were mixed with 100 μl of Nucleofector solution R (Amaxa, GmbH) and 1 μg of siRNA duplexes was added. Using the Nucleofector program T-09, a specific electrical current is applied to the cells and the DNA is delivered into the nucleus. Cells were transferred to culture dishes containing RPMI media supplemented with 20% FBS to facilitate cell attachment. Media was changed 12 hours after transfection and RNA was extracted from cells after 24 hours. Hes6 siRNA duplexes were purchased from Dharmacon (Lafayette, CO). Hes6 siRNA was designed against the human mRNA of Hes6 (GenBank accession number NM_018645) and consists of two selected siRNA duplexes. The target sequence for the duplex 1 was CAGCCTGACCACAGCCCAA (sense: CAGCCUGACCACAGCCCAAUU; antisense: 5'-P UUGGGCUGUGGUCAGGCUGUU) whereas the target sequence for duplex 2 was AAGCTTGAACTTGCCACTTCA (sense: r(GCUUGAACUUGCCACUUCA)dTT; antisense sequence: r(UGAAGUGGCAAGUUCAAGC)dTdT).

## Material collection

### Patients

A database encompassing patients undergoing radical prostatectomy at Addenbrookes Hospital, Cambridge was interrogated. Sections containing regions of normal, BPH, PIN and cancer were classified by uro-pathologists within specimens taken from 32 patients. The cancerous regions were further sub-divided into Gleason Grade 3,4 and 5. Duplicate cores were taken from within each section to provide material corresponding to each Gleason Grade as well as BPH, PIN and Normal material for each patient. This material was arrayed on a tissue microarray (TMA). Material was collected with full ethics approval from a local ethics committee (MREC/01/4/061 and LREC 02/281 M) and as part of the ProMPT Study: Molecular mechanisms and the development of novel treatment strategies in progressing prostate cancer – Northern (and Bristol) Prostate Cancer Collaborative).

### Mice

Mouse materials were collected and processed as described earlier [15]. This was conducted in compliance with international guidelines as confirmed in the original paper describing the model.

### Tissue Microarray

Paraffin blocks from identified patients that had been selected for construction of a tissue micro-array (TMA) were cut using a standard microtome at 5 μm thickness and stained with hematoxylin and eosin (H&E). Confirmation of tissue status (Gleason grades and BPH) was conducted by an uro-pathologist, who assessed and marked the blocks appropriately. 0.6 mm tissue cores were cut and constructed according to pre-determined layout.

### Immunohistochemistry

For NT (Sigma), NTR2 (Acris Antibodies GmbH) and Ascl1 (Aviva Systems Biology) antibodies, antigen retrieval was performed in Tris-EDTA at pH 9.0 in a microwave for 15 minutes. Blocking was performed using 1% donkey serum in PBS for 1 hour. Primary antibody dilutions were as follows: NT and Ascl1 were used at 1:100 and NTR2 at 1:500. Staining was performed using the same blocking solution at 4°C overnight. After three washes in PBS, a biotin-SP-AffiniPure donkey anti-rabbit secondary antibody was applied at a dilution of 1:200 for 45 minutes. Visualization was achieved using an VECTASTAIN Elite ABC kit (Vector Laboratories) for 45 minutes and colour was accomplished using 3,3'-diaminobenzidine (DAB) for 1 minute.

### Immunostaining assessment

Following immunostaining protocols, the TMAs were assessed to determine the degree of TMA core loss or disruption, which varied between the different TMAs. In those cores that remained intact, immunostaining was evaluated according to staining intensity. Scoring was performed independently by two observers (one an independent specialist uro-oncology pathologist) both blinded to the TMA plan. Staining intensity for Ascl1, NTR2 and NT were scored on a scale of 0–5, where 0 means no staining, 1 means minimal staining and 5 means maximum intensity. For clarity of presentation, the staining was then classified into negative (0), low (1–2), medium (3), high (4) and very high (5) intensity. The two assessors compared scores and a consensus agreement was reached on the staining intensity of each core.

## Results and Discussion

**Figure 1:**
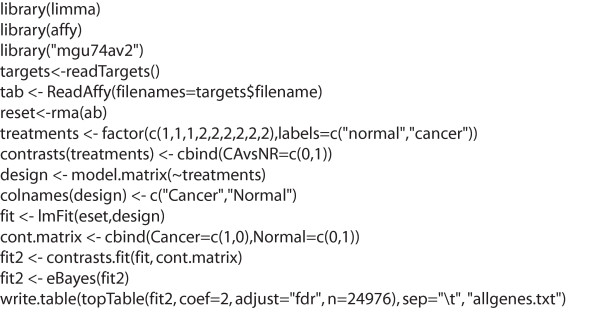
**Script used to identify differentially expressed transcripts in expression array data for the prostate *p53*^PE-/-^; *Rb*^PE-/- ^mouse model**. Data were pre-processed using the RMA (Robust Multichip Average) method, before fitting a linear model and applying Bayesian smoothing to identify differentially expressed genes between the normal and cancer samples.

**Figure 2:**
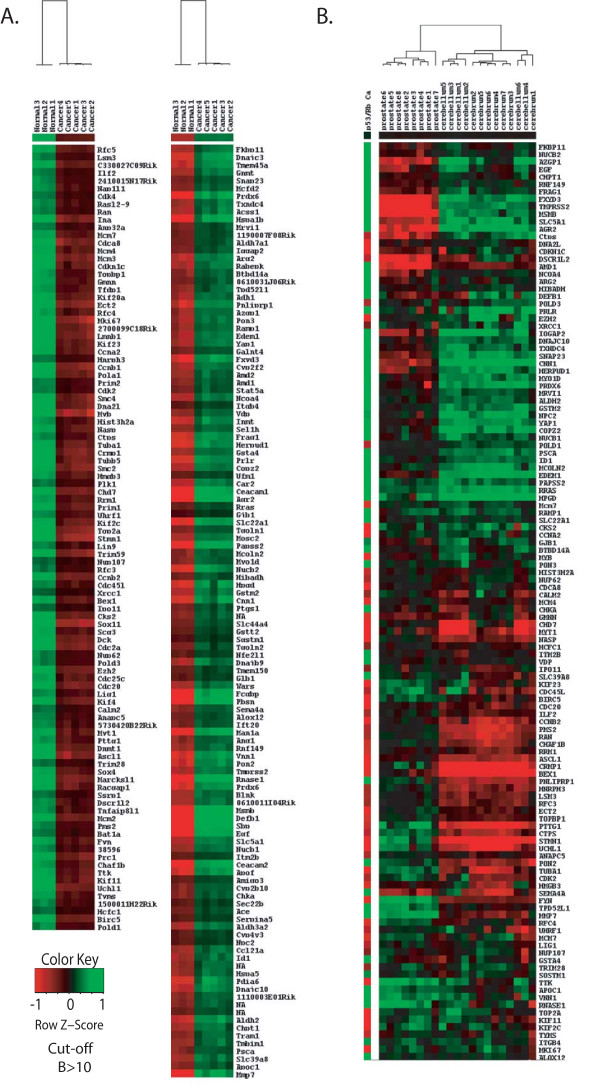
**Over-expressed transcripts in a conditional mouse model for metastatic prostate cancer coincide with those expressed in normal cerebrum/cerebellum adult tissues**. **(a) **Hierarchical clustering of gene expression profiles from normal and prostate cancer tissues in *p53*^*PE*-/-^; *Rb*^*PE*-/- ^mice showing clusters of up and down-regulated genes. Heatmap colours represent relative mRNA expression as indicated in the colour key. **(b) **A heat map generated by hierarchical clustering of normal prostate, cerebrum and cerebellum adult tissue gene expression (Oncogenomics data) for genes identified as differentially expressed in the *p53*^*PE*-/-^; *Rb*^*PE*-/- ^prostate cancer mouse model. Differentially expressed genes in prostate cancer tissue from the *p53*^*PE*-/-^; *Rb*^*PE*-/- ^mouse coincide with those of normal adult tissue from cerebrum and cerebellum and not from the normal prostate.

We have previously shown that prostate cell-lines selected for resistance to anti-androgens develop a neuroendocrine phenotype [4]. In the present study we attempted to identify the transcriptional drivers of this neuroendocrine signature. To do this we interrogated an expression array dataset from a prostate-specific Cre-LoxP *p53*^PE-/-^; *Rb*^PE-/- ^mouse model of prostate cancer which has neuroendocrine characteristics and which maintains androgen receptor expression [15] (Figure 1). This material consisted of expression data from five metastatic prostate epithelium tumours from *p53*^PE-/-^; *Rb*^PE-/- ^mice and three normal prostates from normal non-recombinant littermates. Two hundred and forty genes were differentially expressed with a z-scored significance level of 1 × 10^-8 ^(B > 10). Among those genes, 135 genes were down-regulated and 105 genes were up-regulated in the neoplasms (Figure 2A). As reported in the initial analysis of this array data by Zhou et al [15], within this signature were 3 up-regulated (*Lmnb1*, *Pttg1 *and *Hnrpab*) and 4 down-regulated transcripts (*Myh11*, *Actg2*, *Mylk *and *Cnn1*), showing that our reanalysis of the data successfully identified previously validated transcription changes. Interestingly, there was also a strong correlation between the gene expression changes identified in the *p53*^PE-/-^; *Rb*^PE-/- ^mouse model and gene expression profiles of human metastatic prostate cancer samples (r = 0.52). There was no such correlation when the *p53*^PE-/-^; *Rb*^PE-/- ^mouse model was compared to expression profiles for benign or localised prostate cancer (r = -0.02 and 0.12), suggesting that this model may specifically recapitulate metastatic prostate disease in humans.

**Figure 3:**
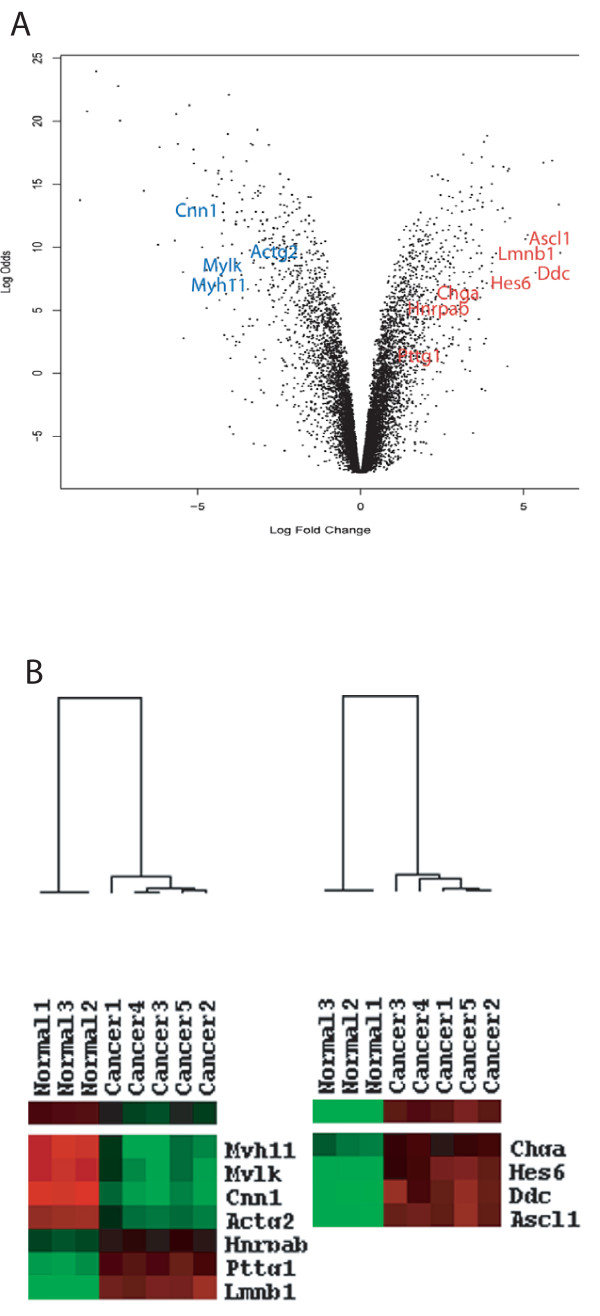
**Pro-neural and neuroendocrine transcripts are over-expressed in the Cre-Lox *p53*^PE-/-^; *Rb*^PE-/- ^knockout mouse model of prostate cancer**. **(a) **Expression array data from the *p53*^PE-/-^; *Rb*^PE-/- ^mouse model is represented in a volcano plot. Transcripts up-regulated are shown in red and those down-regulated are indicated in blue. Pro-neural transcription factors, *Hes6 *and *Ascl1*, and neuroendocrine marker genes such us chromogranin A (*Chga*) and dopa decarboxylase (*Ddc*) are up-regulated in the mouse prostate cancer tumours. Other genes previously shown to be associated with a molecular signature of metastasis in primary solid tumours were also differentially expressed. **(b) **Differentially expressed pro-neural and neuroendocrine genes in *p53*^PE-/-^; *Rb*^PE-/- ^prostate cancers from biological replicate array data are represented in a heatmap (red represents up-regulated genes and green indicates down-regulated genes).

Using data from the Oncogenomics Normal Tissue gene expression Database [19] we assessed the tissue specific expression of the differentially expressed genes. By comparing the complete set of differentially expressed genes obtained for the *p53*^PE-/-^; *Rb*^PE-/- ^mice with this normal tissue expression dataset we observed an enrichment of transcripts concordantly regulated in normal mouse brain and the *p53*^PE-/-^; *Rb*^PE-/- ^tumours (Figure 2B). Taking a stringent cut-off (B > = 10) there was a correlation between the *p53*^PE-/-^; *Rb*^PE-/- ^cancer model and normal brain expression (r = 0.49–0.52). This is in contrast to other normal tissue expression patterns which showed no correlation with the genes differentially expressed in the *p53*^PE-/-^; *Rb*^PE-/- ^model (e.g., the correlation between the *p53*^PE-/-^; *Rb*^PE-/- ^cancer model and normal bladder expression was r = 0.01). This suggests that as these prostate tumours develop they assume some of the gene expression characteristics of neurons (Figure 2B). These changes included the upregulation of two established neuroendocrine markers, *Chga *and *Ddc *(Figure 3B). In addition, two pro-neural bHLH transcription factors *Hes6 *(M = 3.87, B = 7.17) and *Ascl1 *(also called *Mash1*, *Ash1 *and *Hash1*; M = 5.06, B = 10.65) were also up-regulated (Figure 3A–B).

The implication of the expression signature from *p53*^PE-/-^; *Rb*^PE-/- ^mouse tumours is that a transdifferentiation program has been initiated by knocking out *p53 *and *Rb*. This is reflected in the expression of neuroendocrine markers and the change in levels of activator-type and repressor-type bHLH transcription factors. Clearly this does not produce a pure neural lineage in the tumours since they continued to express the androgen receptor together with cytokeratin 5, a basal cell marker, and cytokeratin 8, a luminal epithelial cell marker [15].

**Figure 4:**
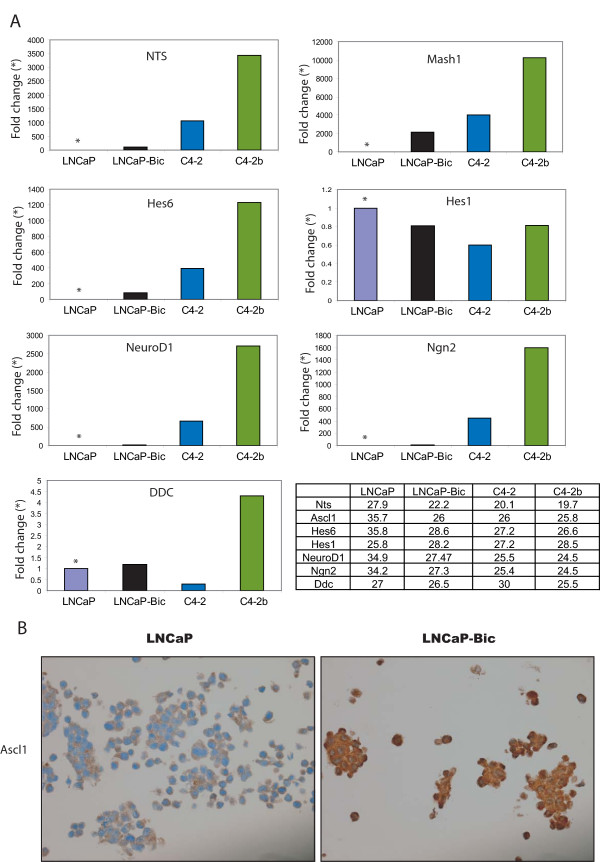
**Pro-neural transcription factor expression is increased in more invasive/metastatic prostate cancer cell-lines**. **(a) **The expression of pro-neural and neuroendocrine transcripts was measured by quantitative RT-PCR using four different cell lines. In the bicalutamide resistant cells (black bars), the androgen-independent cells C4-2 (blue bars) and the androgen-independent metastatic C4-2b cells (green bars) the expression of *Nts*, *Ascl1 *and *Hes6 *with metastatic potential of the lines. The cell line data is presented in order of metastatic potential in a xenograft model of prostate cancer (LNCaP on the left-hand side having limited metastatic potential progressing through to C4-2b with the potential to generate bone metastases upon implantation). Data is represented as fold change as comnpared with the parental LNCaP cell line (light blue bars with asterisk). Also there is expression of *NeuroD1 *and Neurogenin2 (*Ngn2*) in the metastatic cells but to a lesser degree. No changes in the expression of *Hes1 *were observed in any of the cell lines. All the data was normalized using the average of four housekeeping genes (*Gapdh*, *Sdh*, *Ubc *and *Tbp*) and the ratio against the parental line (purple bars) was plotted. A table with the Ct values obtained for each of the genes prior to analysis is provided **(b) **Immunohistochemistry for Ascl1 on paraffin embedded sections from LNCaP and bicalutamide-resistant LNCaP cell-lines.

We sought to test whether this change was also a hallmark of androgen resistance in LNCaP cell-lines that were anti-androgen resistant (LNCaP-Bic), or derived from castrate resistant (C4-2) and osseous metastatic (C4-2b) mouse xenografts. We compared the levels of previously reported markers, neurotensin (NTS) and dopa decarboxylase (DDC), as well activator-type bHLH transcription factors (*Ascl1*, *Hes6*, *NeuroD1 *and *Ngn2*) using real-time PCR and found that they were all elevated in the three resistant cell lines (C4-2b>C4-2>LNCaP-Bic) compared to the parental LNCaP cells (Figure 4A). For instance, *Nts *was 3500 fold higher in C4-2b than the parental LNCaP cell line, *Hes6 *and *NeuroD1 *were 1200 and 2600 fold higher respectively, whereas *Ascl1 *was increased 10^4 ^fold. Other members of the neurogenin family of transcription factors including *Ngn1 *and *Ngn3 *were also increased across the cell lines but to a lesser degree than *Ngn2 *(data not shown). The reduced expression of a repressor-type bHLH factor such as Hes1 could account for these changes in the expression of these neural markers, however we found no change in the level of the Hes1 transcript across the different cell lines (Figure 4A). The expression of *Ddc*, an established neuroendocrine marker gene, was higher in the C4-2b cells only. Overall, we found that neural transcripts increased as the cell lines became more aggressive. To examine the expression of Ascl1 and Hes6 at the protein level, paraffin embedded material from parental and bicalutamide resistant cells was used. Hes6 protein was barely detectable in the LNCaP line but was highly expressed in the bicalutamide resistant cells (data not shown). The localization of the protein in the LNCaP-Bic cell line was mainly nuclear although some staining was observed in the cytoplasm (data not shown). Ascl1 was also highly expressed in the resistant cells (Figure 4B).

**Figure 5:**
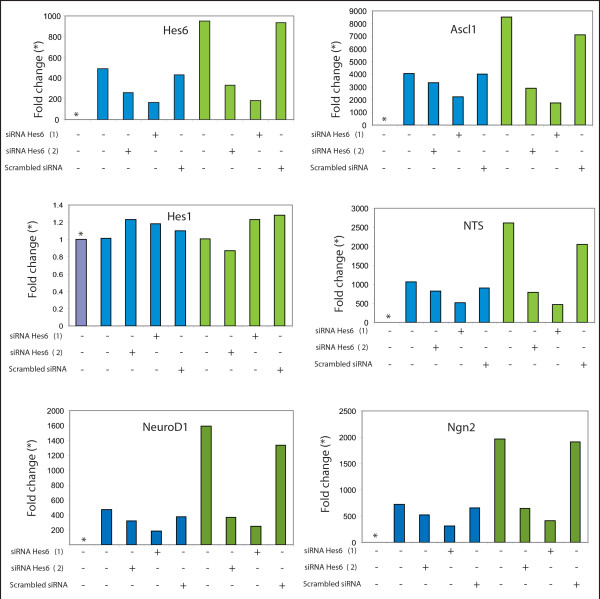
**Hes6 regulates the transcription of other pro-neural factors**. The transcription of *Hes6 *was silenced by siRNA technology using two different duplexes in both C4-2 (dark blue bars) and C4-2b (green bars) cells. Data is represented as fold change as comnpared with the parental LNCaP cell line (light blue bars with asterisk). No effect was observed when the scrambled RNA was used. Other transcripts such as *Ascl1*, *Nts*, *NeuroD1 *and *Ngn2 *were also affected by *Hes6 *knockdown, however no effect was observed when the *Hes1 *transcript was analyzed. As previously reported the levels of pro-neural transcription factors in the C4-2 and C4-2b lines are significantly elevated versus the LNCaP cell-line with only *Hes1 *levels largely unchanged.

Based on reports that Hes6 can regulate the expression and activity of a panel of other transcription factors including Neurogenins and Ascl1, we went on to target Hes6 with two different RNA interference duplexes in two of the resistant cell-lines (C4-2 and C4-2b) (Figure 5). This strategy led to a significant reduction (>80%) of *Hes6 *transcript levels in both lines but also induced the downregulation of *Ascl1*, *Ngn2*, *Nts *and *NeuroD1 *effectively reversing the neuroendocrine phenotype. *Ngn1 *and *Ngn3 *levels were also reduced (data not shown). Hes1 expression was unaffected.

**Figure 6:**
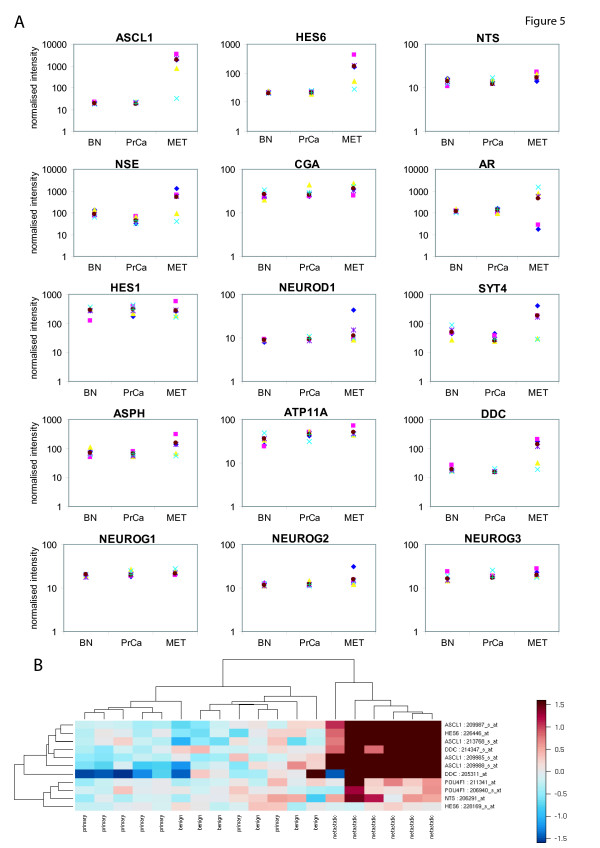
**Pro-neural and neuroendocrine transcripts are over-expressed in metastatic human prostate cancer**. **(a) **The transcription factors *Ascl1 *and *Hes6 *are increased in a small set of metastatic prostate cancers as well as the neuroendocrine markers neuron specific enolase (*Nse*) and dopa-decarboxylase (*Ddc*). Hes1 expression did not change whereas *NeuroD1 *was slightly increased in metastatic tissue. The *AR *expression in metastatic tissue was found to be subdivided in two groups. *NeuroD1 *and synaptotagmin4 (*Syt4*) were also found increased. **(b) **Pro-neural factors discriminate between primary carcinoma and metastasis in prostate cancer. Using clustering analysis, it was possible to discriminate between metastasis and primary tumours by the expression of the pro-neural/neuroendocrine markers *Ascl1*, *Hes6*, *Ddc*, and *Nts *in human prostate tissue.

To determine whether the high expression of the pro-neural transcription factors *Hes6 *and *Ascl1 *in metastatic mouse tumours extrapolated to human diseases, we utilised a publicly available gene expression microarray data set that included six benign, seven primary prostate cancers and six metastatic prostate cancers from human tissue samples [28]. *Ascl1 *and *Hes6 *were highly expressed in all the metastatic tissue samples (p-values 0.001 and 0.01 respectively) (Figure 6A/Table 1). Neuroendocrine markers such as neuron specific enolase (*Nse*) and dopa decarboxylase (*Ddc*) were over-expressed in most of the metastatic samples, with p-values of 0.01 and 0.003 respectively for the transition from prostate cancer to metastasis. Androgen receptor (AR) expression was elevated in half of the metastatic samples and down-regulated in the other half when compared to the benign material (Figure 6A). Previously it has been reported that the AR continues to be expressed during prostate cancer progression and persists in a majority of patients with hormone refractory disease [29,30]. Our observation that the AR is upregulated in some metastatic samples and downregulated in others concords with the finding that in post-mortem metastatic prostate tissue AR expression is more heterogeneous with AR-positive and AR-negative populations of tumour cells between and within the same patient [31].

Once again we were able to rule out derepression of differentiation signals as a contributory factor to the neural phenotype (e.g., through loss of Hes1 expression). *Hes1 *transcript levels remained unchanged in the clinical material in agreement with our observations in the LNCaP cell-line (Figure 6A). A member of the NeuroD family of pro-neural transcription factors *NeuroD1 *was also up-regulated in the metastatic samples (Figure 6A). The neurogenin family of pro-neural transcription factors was also studied. Neurogenin 1 (*Ngn1*) did not significantly change, neurogenin 3 (*Ngn3*) slightly increased in metastatic tissue, and neurogenin 2 (*Ngn2*) was also up-regulated in this tissue (P = 0.03) (Figure 6A). Some of the targets we have previously identified as up-regulated after long-term treatment with bicalutamide were also assessed [4]. Of those, synaptotagmin 4 (*Syt4*) and aspartate beta-hydroxylase (*Asph*) were increased in the transition from localised prostate cancer to metastatic tumours with p-values of 0.006 and 0.001 respectively, whereas *Atp11a *was highly increased in prostate cancer (P = 0.005) (Figure 6A). When a cluster analysis of the same data set was performed, it was possible to discriminate between metastatic and benign and primary tumours by using *Ascl1*, *Hes6*, *Ddc *and *Nts *as marker genes (Figure 6B).

**Figure 7:**
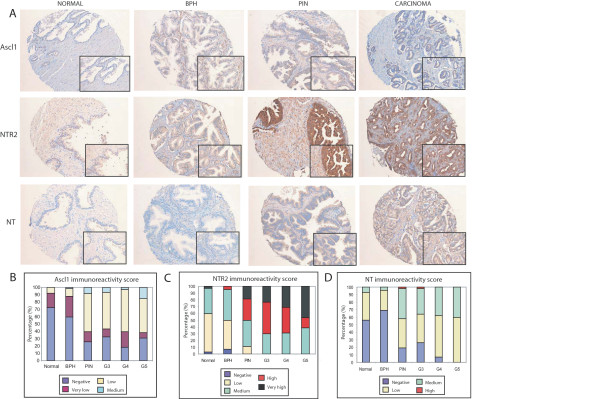
**NTR2 expression increases progressively during the cancer development whereas NT and Ascl1 expression is upregulated at the BPH-PIN transition**. (a) Immunohistochemical analysis of prostate tissue microarrays comprising normal, BPH, PIN and adenocarcinomas. Sections were stained with anti-NT, anti-NTR2 or anti-Ascl1 antibodies. NTR2 immunoreactivity is most intense in PIN and carcinomas, in contrast NT and Ascl1 immunoreactivity is mostly observed in the carcinomas. (b-d) Histopathological score for each specific prostate tissue based on the immunoreactivity antibody staining.

We next sought to test the validity of Ascl1, neurotensin receptor (NTR2) and NTS as cancer markers in clinical prostate material from a distinct cohort of patients by immunohistochemistry (Figure 7A). NTS and Ascl1 were negative in around 70% of normal specimens becoming detectable in around 70% of samples on the transition to PIN and maintaining this expression level throughout subsequent Gleason grades (Figures 7B and 7D). NTR2 by contrast was detectable in around 95% normal samples at medium to low levels. NTR2 staining intensity increased progressively with tumour grade with moderate to very high expression levels in 100% of samples at Gleason Grade 5 (Figure 7C).

**Figure 8:**
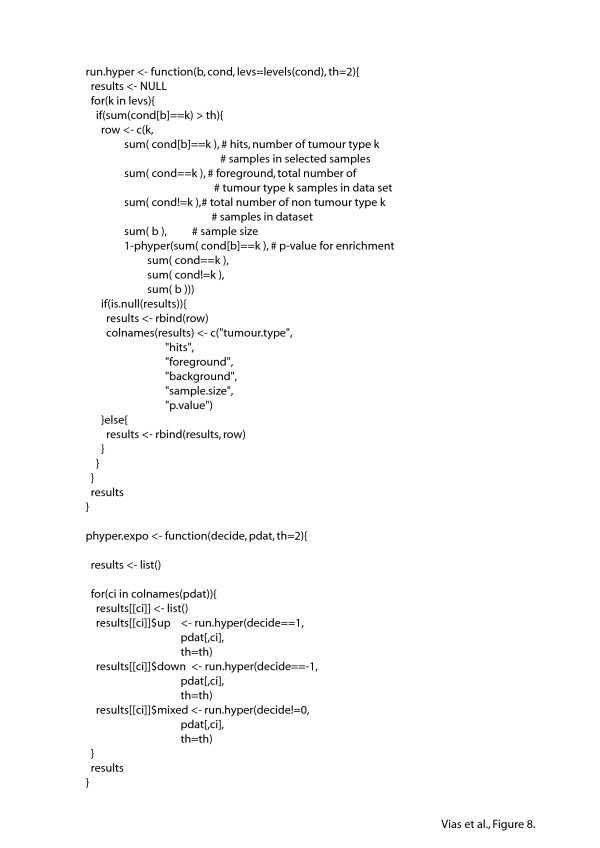
**Script used to test the ability of pro-neural and neuroendocrine transcripts to segregate tumours based on organ site from a multi-tissue cancer expression array dataset**. The DBI package within R was used to extract neuroendocrine gene profiles (205311_at:DDC, 206291_at:NTS, 206940_s_at:POU4F1, 209985_s_at:ASCL1, 209987_s_at:ASCL1, 209988_s_at:ASCL1, 211341_at:POU4F1, 213768_s_at:ASCL1, 214347_s_at:DDC, 226446_at:HES6, 228169_s_at:HES6, 204697_s_at:CHGA). Each gene profile was scaled to it's median value across all samples and a Bioconductor Expression Set object created. The profiles were grouped into functional gene sets.

**Figure 9:**
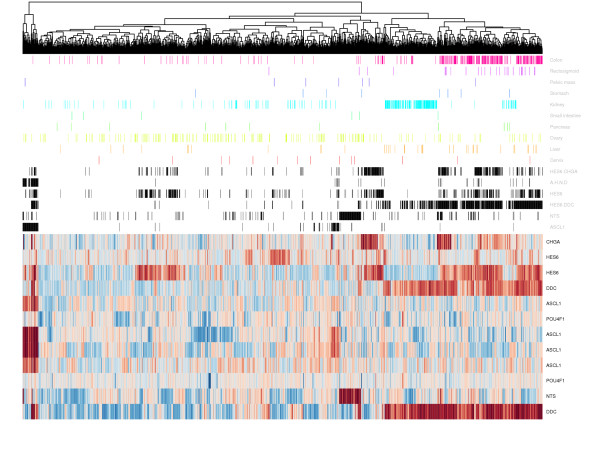
**Subgroups of different cancers can be identified using a proneural signature**. Expression vectors of neuroendocrine biomarkers across the 1786 sample ExpO data set are shown in the heatmap. This was generated using the heatmap function within Bioconductor. Each gene is scaled to its median intensity across all samples. Samples displaying a greater than 3 fold increase in expression for each of the neuroendocrine gene sets are highlighted with black bars above the heatmap. These samples were tested for enrichment of malignant tissue types (**Table 3**). Malignant tissue samples enriching proneural signature sample groups are highlighted as coloured bars at the top of the heatmap.

Ascl1 has previously been reported to be over-expressed in small cell lung cancer, medullary thyroid tumours and astrocytomas, amongst others. There has however been no attempt to explore the power of these markers in clustering tumours at different organ sites from material collected and array profiled in a standardised manner (Figure 8). The expression project for Oncology (ExpO) at Gene Expression Omnibus provides that possibility by making publicly available the complete raw expression array datasets for 1786 multi-tissue tumours specimens [32]. Using *Ddc*, *Nts*, *Hes6*, and *Ascl1 *and combinations of these transcripts we were able to segregate this large clinical collection in a statistically significant manner by tissue site (Figure 9/Table 3). This implies that, in addition to prostate cancer, these proteins may have some utility as novel markers of colon, uterus, rectum, liver, endometrium and breast. However, this requires further validation.

**Table 3:** Combinations of neuroendocrine biomarkers are enriched in specific malignant tissue types.

**Hes6**	**tumour. hits**	**Fgr**	**Bgr**	**p. value**	**Reference**
Colon	93	252	1534	2.74E-014	[26, 38]
Uterus	39	94	1692	3.24E-008	-
Rectosigmoid	12	27	1759	0.00043528	-
Rectum	12	32	1754	0.00303994	-
**Hes6/DDC**	**tumour. hits**	**Fgr**	**Bgr**	**p. value**	**Reference**
Colon	197	252	1534	0	-
Rectosigmoid	21	27	1759	1.42E-009	-
Rectum	23	32	1754	5.03E-009	-
Liver	24	38	1748	1.65E-007	-
Kidney	75	214	1572	0.00040486	-
Stomach	5	8	1778	0.00476289	-
**Hes6/CHGA**	**tumour. hits**	**Fgr**	**Bgr**	**p. value**	**Reference**
Colon	89	252	1534	0	-
Uterus	42	94	1692	6.80E-013	-
Endometrium	30	71	1715	6.50E-009	-
Rectosigmoid	13	27	1759	1.02E-005	-
Rectum	13	32	1754	0.00011383	-
Stomach	4	8	1778	0.00324489	-
**NTS**	**tumour. hits**	**Fgr**	**Bgr**	**p. value**	**Reference**
Lung	36	108	1678	1.29E-010	[39]
Ovary	32	170	1616	0.00143643	[40]
Small intestine	3	6	1780	0.00226141	[41]
**Ascl1**	**tumour. hits**	**Fgr**	**Bgr**	**p. value**	**Reference**
Breast	40	317	1469	8.27E-008	-
Lung	20	108	1678	3.76E-007	[42, 43]
**Ascl1/Hes6/NTS/DDC**	**tumour. hits**	**Fgr**	**Bgr**	**p. value**	**Reference**
Lung	15	108	1678	3.79E-006	-
Rectosigmoid	4	27	1759	0.00421239	-

A hypergeometric distribution was used to generate the p-values for each present tumour type. Tumour types mapping to less than 3 samples per group have been filtered out. References included do mention studies that relate the biomarker/s with human carcinoma tissue and most of them include very small sets of samples.

Neuroendocrine biomarkers enrich for specific malignant tissue types in a multi-tumour dataset. Samples from the ExpO expression dataset were clustered based on the expression of neuroendocrine genesets (Hes6, Hes6-Chga, Hes6-Ddc, Ascl1, Ascl1-Hes6-Nts-Ddc). Clusters were defined by a median expression level with a threshold level three-fold greater than the average intensity across all samples. These sample groups were tested for enrichment of malignant tissue type when compared to the complete dataset as background. The p-values were calculated using a hyper-geometric distribution and represent the probability of obtaining the reported level of enrichment had the samples been selected at random.

## Conclusion

In conclusion, distinct prostatic tumour models and material (cell-lines derived from human tumours, transgenic mouse tumours and patient samples) all display the hallmarks of neural transdifferentiation during the progression to metastatic disease which was associated with a change in the balance of activity and expression in favour of activator-type bHLH transcription factors including Hes6 and Ascl1. Similar changes are discernible in subgroups of tumours at other sites. Collectively this suggests that impairing the activation of pro-neural transcription factors may pay dividends in cancer treatment. However, transcription factors are not conventionally druggable. Nonetheless, antisense oligonucleotide therapy has recently entered phaseII clinical trials to target a chaperone protein, clusterin [33]. Suicide gene therapy has also been proposed in which a therapeutic gene, for example Herpes Simplex Thymidine Kinase or E. Coli purine nucleoside phosphorylase, under the control of promoters for transcription factors exclusively over-expressed in cancer cells such as Ascl1 is expressed and activates Ganciclovir to induce cell cycle arrest [34-36]. In addition 'stapled' peptides are being developed to disrupt protein-protein interactions which may become relevant for targeting transcriptional complexes as well [37]. In light of this study and in combination with these new technologies we may, in future, be capable of exploiting transcription factor activity to control cell fate and improve patient survival.

## Competing Interests

The authors declare that they have no competing interests.

## Authors' contributions

MV undertook the real-time PCR, siRNA, assisted in the array analysis and contributed to the writing of the manuscript. CEM analysed the expression array data from the Cre-Lox p53/Rb mice. PE undertook the analysis of the expO dataset. HS worked on the immunohistochemistry. AW reviewed the original radical prostatectomy histological sections and selected tissue areas for inclusion in the tissue microarrays and scored the tissue microarray immunohistochemistry sections. AYN and ZZ developed Cre-Lox p53/Rb prostate cancer model and provided the expression array data for the mice. DEN contributed to the writing of the manuscript. IGM conceived and designed the study, supervised the laboratory work and contributed to the writing of the manuscript. All authors read and approved the final manuscript.

## List of Abbreviations

Ascl1: Achaete-scute complex homologue 1; BPH: benign prostatic hyperplasia; DDC: Dopa decarboxylase; GAPDH: glyceraldehyde 3-phosphate dehydrogenase; Hes: Hairy Enhancer of Split; NTS/NT: Neurotensin; NTR2/NTSR2: Neurotensin Receptor 2; PIN: prostatic intraepithelial neoplasia; RT-PCR: real-time polymerase chain reaction; SDH: succinate dehydrogenase; TBP: TATA binding protein; UBC: ubiquitin C.

## Pre-publication history

The pre-publication history for this paper can be accessed here:


